# Exploring the link between the pediatric exposome, respiratory health, and executive function in children: a narrative review

**DOI:** 10.3389/fpubh.2024.1383851

**Published:** 2024-10-16

**Authors:** Cecilia S. Alcala, Jamil M. Lane, Vishal Midya, Shoshannah Eggers, Robert O. Wright, Maria José Rosa

**Affiliations:** ^1^Department of Environmental Medicine and Climate Science, Icahn School of Medicine at Mount Sinai, New York, NY, United States; ^2^Department of Epidemiology, University of Iowa College of Public Health, Iowa City, IA, United States; ^3^Institute for Climate Change, Environmental Health, and Exposomics, Icahn School of Medicine at Mount Sinai, New York, NY, United States

**Keywords:** pediatric environmental health, asthma, executive function, respiratory health, exposome

## Abstract

Asthma is a highly prevalent inflammatory condition, significantly affecting nearly six million U.S. children and impacting various facets of their developmental trajectories including neurodevelopment. Evidence supports a link between pediatric environmental exposures in two key areas: asthma and executive function (E.F.). E.F.s are a collective of higher-order cognitive processes facilitating goal-oriented behaviors. Studies also identify asthma-associated E.F. impairments in children. However, limited research has evaluated the inter-relationships among environmental exposures, asthma, and E.F. in children. This review explored relevant research to identify and connect the potential mechanisms and pathways underlying these dynamic associations. The review suggests that the role of the pediatric exposome may function through (1) several underlying biological pathways (i.e., the lung-brain axis, neuroendocrine system, and hypoxia), which could drive asthma and maladaptive E.F. in children and (2) the relationships between the exposome, asthma, and E.F. is a bidirectional linkage. The review reveals essential synergistic links between asthma and E.F. deficits, highlighting the potential role of the pediatric exposome.

## Graphical abstract

This diagram illustrates the underlying pathways between the exposome, lungs, and E.F. The lungs and the brain are interconnected through neuro, endocrine, and immune signaling pathways. Exposomic factors affect the lungs, which can cause asthma, and this, in turn, affects E.F. through metabolic signaling pathways that send metabolic signals from the lungs to the brain.

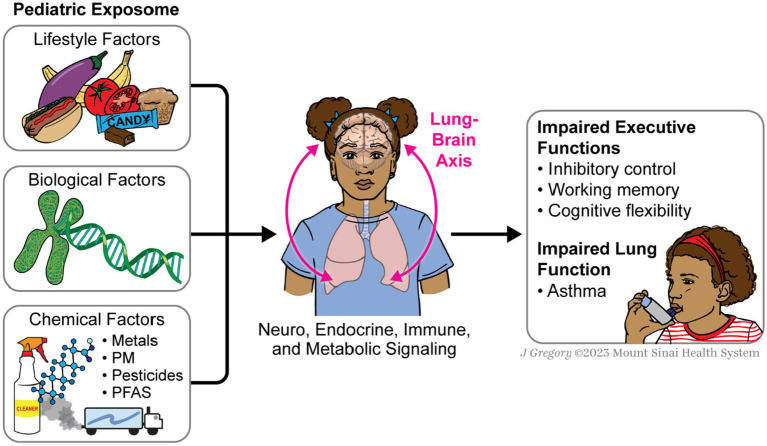

## Highlights

- Accumulating scientific evidence supports a link between environmental exposures and asthma and executive function, respectively, and a link between asthma and executive function in children.
- There is a gap on the evaluation of the complex synergistic link between environmental chemical exposures, asthma, and E.F. outcomes in children.
- This review explores relevant research to identify and connect the potential mechanisms and pathways underlying these dynamic associations.
- We will focus on the chemical exposure due to its heightened risk for children’s health.

## 1 Overview of the exposome and pediatric health

The exposome represents a comprehensive assessment of the environment (1) including all the chemical and non-chemical exposures an individual encounters throughout their life course and how these exposures influence human development and health (2, 3). The exposome can be divided into two domains although there is considerable overlap in the 2 domains: (1) external and (2) internal exposome (4, 5). External refers to exposures external to the human body involving chemical, physical, and psychosocial mechanisms such as environmental contaminants (e.g., heavy metals, pesticides, and other persistent organic pollutants) and non-chemical environmental exposures (e.g., diet, stress, climate, infections, among others). Internal refers to the biological processes within an organism involving molecules, internal chemical components (such as endogenous metabolites and exogenous chemicals derived from the environment), and biological reactions to external exposures (2). Comprehensively identifying environmental factors affecting pediatric health and well-being requires understanding the exposome. Investigating the exposome offers an opportunity to explore multiple ecological layers affecting health, including environmental biomarkers, biological mechanistic pathways, and adverse health outcomes, particularly during sensitive and critical developmental stages of life, as it provides researchers and scientists with invaluable insights to explore causal interactions among and between the exposome for formulating effective prevention strategies.

Environmental exposures begin *in-utero* and can interact with various developmental processes including genetics, epigenetics, and physiological mechanisms starting prenatally through childhood. Specifically, the exposome is critical as the early years of life are highly susceptible to increased physiologic disruptions, increasing the risk for exposure-associated health outcomes that can have long-term effects (6). Evidence has shown that the pediatric exposome involving chemical exposures such as essential and nonessential metals, per-and poly-fluoroalkyl substances (PFAS), pesticides, phthalates, and air pollution has been associated with a range of adverse developmental health outcomes, including respiratory disease and maladaptive cognitive development. Due to children’s distinctive biological composition and behaviors, environmental exposures are more hazardous and detrimental to their health than adults. Childhood is a time of rapid physiologic growth and development, during which various biological systems and organs are at different maturation stages, often with underdeveloped detoxification mechanisms (7, 8). Moreover, children suffer from greater exposure levels as they consume more environmentally contaminated substances relative to their body weight than adults. They also engage in unique behaviors that introduce them to different routes of exposure, including spending more time outdoors (9, 10) and engaging with different consumer products (11, 12). This increased vulnerability is concerning, especially with the rising occurrence of environmental exposure-related diseases like asthma and cognitive impairments in executive functions (E.F.s).

Accumulating scientific evidence supports a link between environmental exposures and (1) asthma and E.F.s and (2) a link between asthma and E.F. in children, but little to no research has evaluated the complex synergistic link between environmental chemical exposures, asthma, and E.F. outcomes in children. This review primarily explores relevant research to identify and connect the potential mechanisms and pathways underlying these dynamic associations. Our focus is directed toward chemical exposure due to its heightened risk for children’s health. Thus, this narrative review will (1) provide a comprehensive review of the literature on the relationships between chemical exposures on (a) respiratory disease and (b) E.F.s; (2) leverage the literature to investigate the (c) link between asthma and E.F. dimensions and (d) the dynamic role of the exposome in the underlying pathways between asthma and E.F.s; and (3) conclude with an discussion on the needs regarding future research in this area.

## 2 Chemical exposures and respiratory disease in children

Respiratory diseases are associated with significant global mortality and morbidity, especially among infants and young children (13). Asthma is one of the most common chronic diseases in children worldwide (14–17). Globally, approximately 300 million people have asthma, and an estimated 100 million more people, mostly children, will have asthma by 2025 (18, 19). In the U.S., asthma and wheezing disproportionately affect specific groups, including children from urban, low-income, or racial/ethnic minoritized backgrounds. Moreover, socioeconomic status is inversely related to lung function (20). Early-life lung function is also an essential predictor for peak lung function in adults, as well as later-life declines in lung function (21, 22). Lung growth correlates with lung function during childhood and adolescence (23, 24) and predicts later-life respiratory disease (21, 24), early multi-morbidity (25), and premature mortality (25, 26).

Prenatal environmental exposures have been associated with respiratory diseases and can potentially alter lung development and growth in childhood and adulthood (27, 28). Lung development begins *in utero* through a carefully orchestrated sequence of events vulnerable to disruption by environmental insults leading to alterations in lung development, affecting the structure and function of the respiratory system across the life span. Therefore, identifying early life risk factors that may be amenable to intervention can provide mechanistic information to better elucidate the link between environmental exposures and childhood respiratory disease and inform the timing of public health interventions.

The fetal environment has been shown to be an unrecognized contributor to disease risk (29, 30), including lung disease (31–34). Lung development begins prenatally through a carefully coordinated sequence of events vulnerable to disruption by even relatively low-dose environmental exposures, which can alter the maturation of organ systems later in life (35). During the first half of gestation, the development of bronchi occurs along with the branching of the airways (36). Throughout the second half of gestation, the alveoli develop, and the lungs continue to mature in number, size, and complexity of the alveoli years after birth (37). Classifying modifiable early life risk factors can illuminate the mechanisms linking environmental exposures to childhood respiratory disease, paving the way for targeted interventions (36). To further explore these mechanisms, the following section investigates the associations between specific chemical exposures and respiratory health outcomes among children. This builds on modifiable early life risk factors outlined in this section.

## 3 Associations between chemical exposures and respiratory health outcomes

### 3.1 Metals

Metals, specifically manganese (Mn) and lead (Pb), have been associated with adverse respiratory health outcomes and lung function deficits in children (36). Mn and Pb are the most common metals individuals are exposed to. Although not well studied in children, Cd is a well-known respiratory toxicant from occupational exposure. These metals are naturally occurring and have anthropogenic sources (36, 38). There is increasing evidence that suggests that prenatal metal exposures adversely affect the subsequent development of allergic diseases in children (39–43). Prenatal metal mixture exposure was associated with childhood allergic rhinitis (39). Prenatal lead was associated with an increased risk for atopic sensitization to common aeroallergens in children at 5 years of age in Poland. These results suggest that low levels of *in-utero* lead exposure may be implicated in the process of allergic sensitization (44). In Italian adolescents, personal air manganese levels were associated with greater odds of reported asthma and asthma medication use (45). Exposure to lead has also been associated with lung function deficits in children (46–48). In Mexico City, researchers found associations between prenatal Pb and childhood lung function were modified by disrupted maternal cortisol in pregnancy and child sex (49).

### 3.2 Per- and polyfluroalkyl substances

Per-and polyfluroalkyl substances (PFAS) are industrial chemicals used in numerous commercial products, which include fabric, cookware, and food container coatings (50). They are also used for aqueous film-forming foams used in firefighting (50–53). PFAS bioaccumulates with the highest concentrations detected in the liver and blood (50). It has been phased out of productivity in the United States, and alternative PFAS has taken its place. When PFAS is inhaled, it targets the lung, and it is known to modify lung surfactant function and proinflammatory responses (50, 54–58). Previous studies have reported associations between exposure to PFAS and asthma, airway hyper-responsiveness (AHR), and inflammation (50, 59–61). There have been inconsistencies (62, 63) in the studies reporting positive associations between exposure to PFAS and asthma-related outcomes in children (64, 65). In Norway, total PFAS serum concentrations were associated with the occurrence of asthma (50, 66) Total perfluorooctane sulfonic acid (PFOS), linear PFOS, and linear perfluorohexane sulfonate (PFHxS) doubled the odds of asthma (50, 66). A correlation was found between recurrent respiratory tract infection and perfluorobutane sulfonic acid (50, 67). PFAS exposure likely alters lung biology influencing asthma, and airway hyper-responsiveness (50).

### 3.3 Phthalates

Phthalates are a group of ubiquitous endocrine-disrupting chemicals used as plasticizers in various industrial commodities and as additives in cosmetics (68, 69). They are used in cosmetic and personal care products, food packaging, toys, containers, shower curtains, and cleaning and building materials (70, 71). Phthalates with low molecular weight are commonly used in solvents, medications, alcohol denaturants, and fixatives (70, 72). Phthalates can accumulate at measurable concentrations in various environments, which include fresh water, soils, atmosphere, sediments, and landfills (69). Individuals are exposed to phthalates daily through diet, inhalation, breastmilk, and skin contact (69). Phthalate metabolites can also cross the placenta and have been detected in cord blood, amniotic fluid, placental tissue, and neonatal meconium (69, 73). Due to the ubiquitous nature of phthalates in daily life, human exposure to phthalates has become a major concern, specifically among potentially vulnerable populations, including pregnant women and infants (74). There is evidence from both animal (75) and human studies (76, 77) that phthalates affect lung health. In animal studies, *in utero* exposure to phthalates, specifically to di-(2-ethylhexyl)- phthalate (DEHP), has been shown to impact alveolarization, including lack of formation and maturation of the distal parts of alveoli in the lungs of newborn rats exposed to phthalates prenatally (78). Epidemiological studies have described associations between prenatal phthalate exposure and childhood respiratory, allergic, and atopic diseases (70, 74, 79–83). Jahreis et al. reported that maternal urinary concentrations of butyl benzyl phthalate during pregnancy were associated with increased asthma risk at 6 years of age in Germany (82). Gascon et al. reported that prenatal exposure to bisphenol A (BPA) and high molecular weight phthalates increased the risk of wheezing and asthma outcomes in children from birth to 7 years of age in Catalonia, Spain (81). However, Johnk et al. investigated associations between maternal urinary concentrations of phthalate metabolites and asthma, eczema, and rhinitis in offspring aged 5 years and did not report any significant findings in the Odense Child Cohort, a prospective Danish birth cohort (83).

### 3.4 Pesticides

Pesticides are a wide group of chemical substances used to kill or control pests and are used for both large and small-scale farming (84, 85). Individuals are exposed to various pesticides simultaneously; thus, the general population, specifically pregnant women and children, may be exposed to pesticide mixtures (86). A few studies have assessed the associations between prenatal pesticide exposure and adverse children’s respiratory health outcomes, including asthma, wheezing, persistent cough, and lung function impairment. However, the results have been inconsistent (87, 88). Researchers have reported that higher prenatal dichlorodiphenyldichloroethylene exposure is associated with an increased risk of wheeze (89, 90). In addition, higher phosphate metabolite exposure in childhood was associated with decreased lung function at seven years of age among the children of farmworkers in California (91). A study in the Netherlands found no association between children living near agricultural fields likely treated with pesticides and asthma/respiratory outcomes (92). While the importance of investigating mixtures of pesticides is emerging (87, 88, 93), there is a dearth of research investigating prenatal pesticide mixtures and asthma and wheeze, and lung function in childhood (94).

### 3.5 Particulate matter

Particulate matter (PM) is a complex mixture of solids and liquids suspended in the air. The PM can be categorized by its aerodynamic diameter as PM_10_ (<10 μm, inhalable particulate matter), PM_2.5_ (<2.5 μm, fine particulate matter), and PM_0.1_ (<0.1 μm, ultrafine particulate matter) (95). PM_2.5_ is considered the most harmful and can absorb a variety of toxic and harmful substances (96). Due to its small particle size, it penetrates deep into the lungs, deposits in the terminal bronchioles and alveoli with breath, and enters the circulatory system through the gas-blood barrier (97, 98). Exposure to PM_2.5_ can lead to systemic adverse effects, specifically on the respiratory and cardiovascular systems (99). The respiratory system is the primary route of toxicity for PM_2.5_. Individuals are primarily exposed through inhalation (95). Exposure to PM_2.5_ can cause the development and progression of acute and chronic lung diseases, such as tracheal and pulmonary inflammation (100–102), and asthma (103, 104). Moreover, PM_2.5_ exposure may increase the susceptibility to respiratory infections (95).

### 3.6 Summary

Respiratory diseases and respiratory tract symptoms in children are fairly common. Many studies have shown that exposure to chemicals such as pesticides, PFAS, PM, phthalates, and metals can lead to respiratory diseases in children. Most of these studies have reported exposure prenatally as a critical window linked to children developing asthma and wheeze. However, these studies have a few weaknesses, which include sample size or subject selection. Future directions include research on the biological mechanisms of various chemical exposures, individually and as a mixture, and their impact on asthma and wheeze in children. Furthermore, the dissemination of these research findings, and the creation and implementation of effective prevention strategies are essential to increase knowledge of chemical exposures and their impact on adverse respiratory health outcomes in children. In the following section, we delve into children’s E.F.s and their relationship with early-life neurotoxic exposures, comprehensively analyzing the chemistry between these factors.

## 4 Chemical exposures and executive functioning in children

Executive functions (E.F.s) encompass a group of high-level skills that coordinate sophisticated cognitive and behavioral activities and allow for complex functions such as planning, attention, emotional control, organization, focusing on problem-solving, and shifting viewpoints. E.F. development is vital across childhood and consists of at least three central domains – inhibitory control, working memory, and cognitive flexibility. Accumulating scientific evidence suggests that E.F.s are particularly sensitive to early-life environmental neurotoxicants (e.g., trace metals, PFAS, phthalates, pesticides, and particulate matter) as neurotoxicants can penetrate the blood–brain barriers and accumulate in brain regions (e.g., prefrontal cortex, hippocampus, and basal ganglia) that regulate E.F. functionality.

### 4.1 Inhibitory control

*Inhibitory control* is a fundamental E.F. with connectivity within several anatomical brain networks [e.g., caudate nucleus, subthalamic nucleus; (105)], but the prefrontal cortex (PFC) connectivity is essential for regulating and orchestrating successful inhibitory control (106, 107). Inhibitory control enables children to inhibit goal-irrelevant stimuli from interrupting goal-directed behavioral and emotional responses (107, 108), which is critical for cognitive processes relating to attention response, emotional regulation, behavioral control, and social development. Inhibitory control begins to develop rapidly between ages 3 and 6 years (108, 109) and continues until the functional maturation of the frontal lobes, often during late adolescence.

### 4.2 Working memory

*Working memory* is a core cognitive feature of the human cognitive system with underpinnings to the dorsolateral PFC, an area in the PFC during child development (110). It is a system with a temporary capacity for simultaneously processing, holding, and manipulating information (108). Working memory development is vital for children as it is responsible for multi-dimensional cognitive activities such as reasoning, decision-making, problem-solving, comprehension, and learning (111) that are central for everyday tasks while involving dual-task performance with several other high-level cognitive faculties (112). Working memory develops during infancy and dramatically increases during middle childhood (ages 4 to 8), with gradual developments during early adolescence.

### 4.3 Cognitive flexibility

*Cognitive flexibility* is a core executive dimension that amplifies on the first two E.F. domains, primarily supported by several PFC sub-regions (e.g., orbitofrontal, anterior cingulate) but is also supported by subcortical structures such as the posterior parietal cortex and basal ganglia (113, 114). Cognitive flexibility involves focusing on changing perspectives, shifting attention, creative thinking, and adapting flexibly to changeable environments (108, 115). It aids in managing multiple tasks, fostering innovative, adaptable behavior, and fundamentally promoting a range of life outcomes, including academic achievement, career attainment, and successful social relationships (116). During preschool years, cognitive flexibility matures swiftly and progresses throughout adolescence, paralleling the maturation of neural networks encompassing the PFC, while it only reaches full maturation by the early 20s.

## 5 Associations between neurotoxicant exposures and executive function development

Accumulating evidence indicates E.F. can be impaired when children are exposed to various environmental neurotoxicants. As previously mentioned, once in the body, early-life neurotoxicants can cause biochemical neurologic damage by readily penetrating the child’s blood–brain barriers, accumulating in supportive brain regions, disrupting neurotransmitter systems and their activities, and altering structural and functional subcortical components that regulate E.F.s (117, 118). In this section, our attention will be directed toward an assortment of neurotoxicants, including metals, PFAS compounds, particulate matter (PM), pesticides, and phthalate metabolites.

### 5.1 Metals

Environmental epidemiological studies have shown that essential (manganese [Mn], iron [Fe], chromium [Cr], selenium [Se], and zinc [Zn]) metals are critical for child growth and brain development. However, imbalanced metal levels can lead to deficiencies or neurotoxicity, causing maladaptive E.F (119). In addition, any dose exposure to nonessential (arsenic [As], cadmium [Cd], lead [Pb], mercury [Hg], nickel [Ni], and strontium [Sr]) is considered deleterious to E.F. as these metals lack biological significance, with substantial evidence identifying an inverse association between nonessential metals and E.F. performance among children (120–122). Data from children’s studies indicate that prenatal and postnatal under and over-exposure to a panel of essential and nonessential metals can dysregulate E.F. development on an individual exposure (123, 124) and when exposed as mixtures (125–127). For both elements, many studies suggest a sexually dimorphic effect, with E.F. performance differing in the directionality by child sex, depending on the type and dose of metal exposure and time and duration of exposure (128).

### 5.2 Per- and polyfluroalkyl substances

Evidence from human and non-human animal studies suggests that PFAS dysregulates dopamine metabolism (129) while also affecting thyroid function (122). Both processes may have persistent implications for E.F. development. Animal models have demonstrated an increased risk of neurobehavioral deficits relating to inhibitory control mechanisms (130–135). Children’s studies on the neurotoxicity of certain PFAS compounds are limited and inconsistent. Some research investigating prenatal windows of PFAS exposure reported positive associations with cognitive functions underpinned by E.F., such as reading ability (136), attention (137), and memory capabilities (138). Others reported negative associations with measures of response inhibition, working memory, and global E.F.s (139–143). Research has primarily focused on the prenatal effects of PFAS exposure on children’s E.F. outcomes, while the impact of postnatal windows is sparse and overlooked. Most epidemiologic research has focused on investigating a single PFAS compound on cognitive and behavioral measures in children (137, 144, 145), which does not capture the mixed-exposure contexts in which people encounter PFAS. To our knowledge, only one study has examined the mixture effects of PFAS on E.F., but it was conducted in older adults (146). Lastly, in the literature, evidence suggests that there were no consistent patterns of sexual dimorphism in PFAS associations with E.F (147).

### 5.3 Phthalates

Human (148) and non-human animal (149–151) studies suggest that phthalate compounds cause dopaminergic dysregulation, a central neurotransmitter system regulating E.F. and reward processing. Animal models of phthalate exposure demonstrate an increased risk of neurobehavioral deficits (152, 153), and memory impairments (154). Human studies have shown that prenatal phthalate exposures are linked to global E.F.s (155) and cognitive constructs related to E.F., such as delayed psychomotor, language development, intellectual developmental (156, 157), and attention deficits (158, 159), as well as overall abnormalities in cognition and neurobehavior. Some studies have also observed sex-specific effects (155, 160, 161). In addition, most epidemiologic research has focused on investigating a single phthalate compound on cognitive measures (152, 159, 162) with some research discovering a link between mixture effects of phthalates and E.F. development (163, 164).

### 5.4 Pesticides

Pesticide exposures have been linked to E.F. impairment in children (165–167). Several classes (e.g., organophosphorus pesticides [OPPs], carbamate pesticides) of pesticides inherently possess neurotoxic properties by design, acting by directly affecting insects’ nervous systems (168). In children, they have the potential to induce neurological harm (167, 169, 170) even at lower concentrations, manifesting as subtle impacts due to oxidative stress and their influence on fundamental neuronal systems, thereby impairing the dynamics of neurotransmitters involved in synaptic communication (171). Children studies have reported associations between early-life neurotoxic pesticide exposures and poorer E.F. performance in motor inhibition (172, 173), behavioral regulation (173), working memory (173–175), and cognitive flexibility (176, 177). Evidence on the sexually dimorphic effects is limited, yet a few studies report E.F. differences among boys and girls (178–180), highlighting a potentially heightened vulnerability in boys exposed to early-life pesticides.

### 5.5 Particulate matter

Mounting evidence strongly indicates that environmental ambient PM and pollutants associated with traffic could potentially function as agents of developmental neurotoxicity (181, 182). Moreover, findings from various sources, spanning animal experiments to epidemiological research, suggest that the detrimental impacts of PM on E.F. might initiate during fetal development (183, 184). The developing fetal brain is notably vulnerable to the impacts of PM, as multiple studies have shown that PM exposure increases neuroinflammation and oxidative stress (185) and alters brain morphology (186), further establishing the adverse effects of prenatal air pollution on both neurodevelopment and neurobehavioral functions relating to E.F. in children. Past research has illuminated that exposure to various P.M.s, such as particulate matter 2.5 (PM_2.5_) and PM_10_ levels, during distinct sensitive periods like prenatal stages, can amplify the likelihood of maladaptive E.F. trajectories in infants and children (183, 187–190). Limited research has detected sex-specific effects on E.F. outcomes (188), while the majority have not found notable patterns.

### 5.6 Summary

The implications of these findings are substantial, as childhood is a sensitive and pivotal neurodevelopmental stage. Maladaptive E.F. caused by subclinical neurotoxicity during this stage can manifest later in life and yield enduring ramifications, affecting various aspects of life such as quality of life, educational attainment, social interactions, and professional achievements. The intricacies of the temporal relationships between neurotoxicants and E.F. remain unclear, underscoring the need for more longitudinal designs to unravel these temporal dynamics. Despite this uncertainty, it remains imperative to identify and mitigate risk factors associated with neurotoxic exposures. Developing environmental interventions to reduce or eliminate exposure becomes paramount to enhance E.F. development.

## 6 Exploring the link between asthma and executive function

Studies have discovered a link between asthma and E.F. decrements in children (191–193) and adults (194, 195). This linkage may be explained by potential disruptions in the neuroendocrine system and chronic to mild intermittent hypoxia (reduced oxygen levels) in children with asthma (192, 194, 196); thus, leading to less efficient E.F. development. Evidence has shown that asthmatic children manifest difficulties in E.F. tasks relating to shifting attention (191, 193) and emotional and behavioral regulation, mainly when the condition is poorly controlled (197, 198). A cross-sectional study comparing asthmatic and healthy adults using diffusion tensor imaging to investigate whole-brain variations in white matter integrity revealed that adults with asthma exhibited several cognitive deficiencies, including E.F. impairments, which strongly correlated with white matter abnormalities (194). These findings reinforce the notion that children with asthma, particularly those with poorly managed treatments, face a heightened risk of E.F. disturbances (199). Consequently, it becomes imperative to proactively evaluate children’s E.F. capabilities rather than solely focusing on their general intellectual aptitude (191).

Some studies suggest that there may be a bidirectional link between asthma and cognitive activities in children and adults (162, 193, 200), but the data are limited. Asthma may affect cognitive function, including E.F., and poor E.F. may be associated with asthma and other comorbid conditions. Further research is needed to better understand the mechanisms underlying this association and to develop targeted interventions that promote optimal executive functioning in individuals with asthma.

## 7 The role of the exposome in the underlying pathways between asthma and executive function

### 7.1 Neuroendocrine system and hypoxia

The neuroendocrine system is comprised of nerves and gland cells. The purpose of the system is to create hormones and release them into the bloodstream. These cells receive messages and signals from the nervous system and respond by making and releasing hormones. Glucocorticoids and catecholamines are able to modulate various aspects of the immune system. In addition, the pituitary hormones prolactin and growth hormone are also released, which also can modulate the immune system. Conversely, cytokines and hormones released by an activated immune system influence neural and endocrine processes. For example, the sympathetic nervous system innervates immune organs such as the thymus, bone marrow, spleen, and even lymph nodes (201). We noted earlier that stress hormones released during hypothalamic–pituitary–adrenal (HPA) axis activation can adversely impact immune function. One way they do this is by inhibiting the production of lymphocytes, white blood cells that circulate in the body’s fluids that are important in the immune response. Overall, these hormones control numerous body functions (202). Asthma can impact the central nervous system. It can lead to decreases in the oxygen levels due to brain tissue being sensitive (192). During acute asthma attacks the oxygen saturation can drop to persistently low levels which can influence neural abnormalities (192, 203). A potential cause of cerebral hypoxia is severe asthma attacks. These attacks can lead to the loss of consciousness, cyanosis, anoxic brain damage, or even death (192, 204, 205). Previous studies have reported that baseline arterial hemoglobin oxygen saturation can be found in asthma (192, 206–208). Cognitive dysfunction could potentially be caused by neural changes from hypoxia/ischemia in vulnerable brain regions with high metabolic demands (e.g., neocortex, hippocampus, basal ganglia). Additionally, animal studies have examined the effects of asthma- induced intermittent brain hypoxia on learning and memory show deficits, in addition to impaired long-term potentiation (209). Studies have shown chronic asthma could result in multiple cognitive impairments, specifically learning and memory ability. Lastly, chronic asthma damages synaptic structure and function (192, 194, 209).

### 7.2 Lung-brain Axis

It has been recently discovered that the brain communicates with various organs in the body through a neuroanatomical communication system. Studies show a dynamic bidirectional signaling pathway between the lung and brain, referred to as the lung-brain axis, identified through sophisticated metabolomics analysis (210). This bidirectional communication involves several biological components, including the central nervous system (CNS), the autonomic nervous system, and the hypothalamus–pituitary–adrenal (HPA) axis, as well as signaling pathways with metabolites, gases, endocrine, and the immune system (211). Activation of the organ-specific immune system in the lung or the brain can alter the functioning of the respective other organs (212). The lung-brain axis has been explored in various pathophysiological conditions, such as inflammatory and immune components, amyloid-*β* pathology, host–microbe interactions, and ventilator-induced brain injury (213–215). The lung-brain axis is thought to correlate lung microbes to neurodegenerative diseases and changes in behavior (216).

To our knowledge, no human studies have examined chemical exposures’ impact on the lung-brain axis. Much of the research is conducted in rodent models. Evidence suggests that air pollutant exposures in these models lead to serum-borne, cytokine-independent signals that elevate brain proinflammatory conditions connected to the pulmonary response (217). Exposure to zinc oxide nanoparticles may damage the cerebral cortex, possibly via lung-brain axis disruption (218). The lung-brain axis is involved in a process where the pulmonary response to ozone exposures indirectly influences CNS neuroimmune function through circulating and cellular factors (219). Overall, these findings suggest that chemical exposure can impact the lung-brain axis, but the various pathways and mechanisms underlying these effects still need to be fully understood, and further research in humans is needed.

### 7.3 Limitations

Our review have limitations that warrant attention. A key limitation is the exclusive focus on external chemical exposures within the pediatric exposome, omitting internal exposures (2) such as metabolites, as well as non-chemical factors (220) like infections, lifestyle behaviors, psychosocial stressors, and socioeconomic determinants—all of which are integral to the broader exposome framework. Additionally, this review did not consider biological exposures, such as allergens (221), despite their relevance to pediatric health. To comprehensively understand the pediatric exposome, future research should integrate both internal and non-chemical exposures, capturing the multifactorial nature of environmental influences on child health outcomes.

## 8 Gaps and future directions

Most evidence linking asthma to E.F. and the influence of the pediatric exposome comes from animal studies. In human studies, the existing data only examine part of the biological pathways between chemical exposures, asthma, and E.F. There are a number of other ways in which E.F. may influence asthma treatment as well. For example, impulsive behavior may increase risk of exposure to asthma triggers such as allergens, and children with poor cognitive flexibility may delay treatment. Few, if any, human studies have explored whether environmental exposure disrupts the lung-brain axis or how exposures impact pathways between neuroendocrine systems and hypoxia, leading to worse asthma outcomes, which could affect E.F. development. As cognitive development is rapid and critical during childhood, further investigation among human studies on the impact of chemical exposure on the biological pathways between asthma and E.F. is necessary. Epidemiological evidence from robustly designed child cohorts, encompassing diverse variables such as sex, age, and longitudinal follow-up, is imperative. This approach will allow for a comprehensive exploration of all three components within each pathway, leading to deeper insights into the dynamic interconnections. We also emphasize the importance of employing advanced statistical methodologies to better understand the dynamics between exposure, asthma, and E.F.s (222). The literature has primarily relied on traditional regression models that examine individual exposures or chemical mixture models, such as Weighted Quantile Sum Regression (223) and Bayesian Kernel Machine Regression (224), which do not fully capture the underlying structural relationships. Therefore, we recommend using a structural equation-modeling framework (225, 226). This approach can effectively capture the complex interrelationships between the chemical exposome, respiratory health, and E.F. dimensions while managing individual variability inherent in studying complex biological and cognitive processes. By adopting this methodology, we can significantly enhance our comprehension of the intricate causal dynamics underlying these relationships (227, 228). Furthermore, there have been extensive advancements in measuring external exposures with personal wearable device (229, 230). This could be used with other omics measurements and linked to pediatric exposome – health in the future. Utilizing technology advancements in epidemiologic research would be an effective way to measure particulate matter, for example, and assess the relationship between executive function and respiratory health outcomes across the lifespan (231). Additionally, researchers collaborating with health care providers to measure the deficits in cognition and their associations with asthma would be useful. This will provide insight into these associations found during primary care and/or asthma specialty clinical visits.

## 9 Conclusion

This narrative review explored the state of the literature pertaining to the relationship between the pediatric exposome, respiratory health, and E.F. in children. Here, we present scientific evidence that may suggest the role of the exposome through the lung-brain axis, neuroendocrine systems, and hypoxia, which could potentially be the underlying pathways between asthma and E.F. These relationships are not unidirectional but rather bidirectional, as evidence suggests that the lung-brain axis is a two-way communication pathway between the brain and the respiratory system, and the neuroendocrine system is involved through HPA, which integrates cognitive and behavioral responses to early-life exposures. This review implies that human studies in children are needed to understand better the synergistic relationships on the impact of chemical exposure on the biological pathways and interactions between asthma and E.F.
